# Titanium Dioxide in Biomedical and Environmental Nanotechnology: From Photocatalytic Detoxification to Targeted Therapeutics

**DOI:** 10.3390/molecules31071197

**Published:** 2026-04-03

**Authors:** Avraham Dayan, Gideon Fleminger

**Affiliations:** 1 Multidisciplinary Vesicle Program, Life Sciences Core Facilities, Weizmann Institute of Science, Rehovot 76100, Israel; avraham.dayan@weizmann.ac.il; 2 The Shmunis School of Biomedicine and Cancer Research, George S. Wise Faculty of Life Sciences, Tel Aviv University, Tel Aviv 69978, Israel

**Keywords:** titanium dioxide, reactive oxygen species, photocatalysis, metal–support interactions, environmental remediation, photodynamic therapy, implant, nanotoxicology, moonlight proteins

## Abstract

Titanium dioxide (TiO_2_) has evolved from a conventional photocatalyst into a sophisticated nano-platform that bridges environmental sustainability and biomedicine. This paper proposes a unified interfacial redox design framework that links the electronic-structure engineering of the TiO_2_ with the spatial control of its reactive oxygen species (ROS). In the environmental sector, we highlight advances in photocatalytic detoxification, such as the cleavage of organophosphates via Ag-modified TiO_2_, driven by doping and metal–support interactions. In the biomedical domain, TiO_2_ is framed as an active bio-interface capable of coordinative protein binding. We specifically examine the “moonlighting” protein dihydrolipoamide dehydrogenase (DLDH) as a model for stable, oriented biofunctionalization. By integrating RGD-targeting motifs, these hybrid systems enable integrin-directed, localized photodynamic effects. We further address critical toxicological considerations, emphasizing that TiO_2_ behavior is context-dependent and governed by particle size, crystallinity, and surface state. By synthesizing insights from catalysis and redox biology, this manuscript outlines principles for the rational design of safer, application-specific TiO_2_ technologies. This convergence supports a transition from non-selective oxidation toward predictable, spatially confined redox outcomes in both complex environmental matrices and physiological systems. This review outlines key mechanistic insights and proposes design principles for controlled and context-dependent TiO_2_ activity.

## 1. Introduction

Photocatalysts are materials that use light energy to accelerate chemical reactions without being consumed in the process. They are widely used in environmental cleanup (like air and water purification) and energy production (like hydrogen generation). Out of them, titanium dioxide (TiO_2_) is one of the most extensively investigated inorganic nanomaterials due to its high surface activity, chemical stability, favorable electronic-structure, compatibility with diverse chemical and biological environments, and low price [1,2,3,4]. TiO_2_ exists primarily as three phases: anatase, which is generally considered the most photo catalytically active phase; rutile; and brookite [1]. It has a wide bandgap of approximately 3.2 eV, meaning it can only be activated by ultraviolet (UV) light unless in a doped form [5]. Beyond its classical role in heterogeneous photocatalysis, TiO_2_ has evolved into a multifunctional platform that supports controlled redox activity, molecular adsorption, and nano–bio interfacing [3,4,6,7]. A distinctive feature of TiO_2_ is its capacity to engage in coordinative interactions with proteins and metabolites, enabling the emergence of hybrid systems in which biological components exhibit so-called moonlighting functions at inorganic surfaces [8,9,10,11]. This conceptual shift positions TiO_2_ not merely as a catalyst or coating, but as an active participant in physicochemical and biological processes. A central premise of this review is that the same interfacial redox physics governing pollutant mineralization also dictates biological ROS signaling and nano–bio interactions. Recognizing this shared mechanistic core enables cross-disciplinary transfer of design principles. This review aims to unify environmental and biomedical applications of TiO_2_ through the shared framework of interfacial redox chemistry.

The photocatalytic activity of TiO_2_ originates from its semiconductor band structure, which supports photoinduced charge separation and subsequent reactive oxygen species (ROS), including highly reactive intermediates such as hydroxyl radicals (•OH), superoxide anions (O_2_•^−^), and hydrogen peroxide (H_2_O_2_), which collectively govern oxidative reactivity at the interface [12,13,14]. The relative alignment of valence and conduction band edges, defect-state density, and interfacial band bending collectively determine whether photogenerated charges undergo recombination or surface transfer, or participate in radical formation [9,14]. Thus, electronic-structure engineering is not merely a materials science concern but a determinant of biological and environmental outcomes. These processes underpin its widespread use in environmental detoxification, where non-selective oxidative reactions enable mineralization of hazardous organic compounds in air and water [12,13,14]. Catalytic efficiency is strongly modulated by crystal phase composition, surface chemistry, dopants such as single-atom catalysis, and strong metal–support interactions (SMSI), referring to electronic coupling between metal nanoparticles and the TiO_2_ support that modulates charge transfer and catalytic activity [13,15,16,17,18].

Beyond environmental catalysis, TiO_2_ has emerged as an active bio-interface capable of dynamic interaction with proteins, membranes, and cellular signaling pathways [8,19,20]. At the nano–bio-interface, TiO_2_ interacts with biomolecules through coordinative and electrostatic mechanisms that enable stable and oriented binding. Unlike nonspecific physisorption observed for many materials, TiO_2_ can form semi-covalent coordinative bonds with selected biomolecules, enabling stable and oriented immobilization [8,20,21]. Among these, dihydrolipoamide dehydrogenase (DLDH) represents a prototypical moonlighting protein whose surface-binding capacity and redox activity allow it to function as a molecular bridge between inorganic surfaces and biological systems [10,11,22]. Such interactions provide a mechanistic basis for engineering bio functional TiO_2_ constructs with controlled orientation and activity. Here we present DLDH as a mechanistically elucidated example of coordinative bio-interface formation, serving as a model system to illustrate broader principles of protein–oxide interaction [8].

Built on these principles, protein–TiO_2_ hybrids have been explored as platforms for localized redox modulation. Arginine–glycine–aspartic acid (RGD) peptide-modified constructs enable integrin-mediated recognition, while illumination-induced ROS generation provides a basis for spatially confined photodynamic concepts [9,22,23].

This paper integrates environmental and biomedical perspectives by focusing on the common physicochemical framework of TiO_2_ surface reactivity and ROS chemistry. We review advances in catalytic detoxification, nano–bio-interface formation, moonlighting protein interactions, and the associated toxicological considerations. While these developments illustrate conceptual convergence, many of the described systems remain at experimental or preclinical stages and require rigorous validation under physiologically and environmentally relevant conditions. Through this synthesis, we aim to outline design principles for safer, application-specific TiO_2_ technologies bridging sustainability and targeted medical innovation. Specifically, we ask how controlled electronic-structure tuning at the oxide interface can be translated into predictable redox outcomes across environmental and biological systems. This framework provides the basis for the detailed discussion of environmental photocatalytic mechanisms (Section 3) and biomedical applications (Section 5).

## 2. Electronic Structure and Nano–Bio-Interface of TiO_2_

Titanium dioxide occurs primarily in three polymorphs, anatase, rutile and brookite, each characterized by distinct lattice structures and surface energetics [3,4,6,7]. Anatase typically exhibits a wider band gap (~3.2 eV) than rutile (~3.0 eV), a difference that influences light absorption and charge carrier dynamics [12,13,14]. Mixed-phase anatase–rutile systems often display enhanced photocatalytic performance, attributed to interfacial charge transfer that suppresses electron–hole recombination [4,16]. At the nanoscale, TiO_2_ exhibits size-dependent electronic behavior, including altered band bending and increased density of surface-active sites, features that govern interactions with adsorbed molecules and biomacromolecules [1]. Exposure of high-energy facets, particularly anatase (001), further modulates adsorption geometry and redox reactivity, providing a structural basis for application-specific design. At the electronic level, interfacial band alignment between the anatase and rutile phases facilitates directional charge transfer, where electrons preferentially migrate toward rutile while holes remain in anatase domains. This internal heterojunction effect reduces recombination and enhances redox selectivity. Controlled defect engineering, therefore, plays a central role in determining whether TiO_2_ behaves as a sustained ROS generator or as a transient redox mediator, highlighting its relevance for application-specific design.

The surface chemistry of TiO_2_ is dominated by hydroxyl groups, coordinatively unsaturated titanium centers, and oxygen vacancies that collectively dictate adsorption and interfacial reactivity. These features promote strong coordinative interactions with ligands containing carboxylate, phosphate, catechol, and thiol functionalities. Molecular investigations have shown that DLDH binds TiO_2_ through defined coordinative contacts rather than nonspecific adsorption, involving metal-chelating residues within a discrete binding region [8,9,10,23]. From a translational perspective, these interactions provide a basis for designing stable and functionally oriented bio-interfaces with controlled activity. This interaction results in stable orientation and long-term retention of enzymatic and moonlighting functions [11], establishing TiO_2_ as a platform capable of forming semi-covalent bio-interfaces distinct from conventional physisorption layers. TiO_2_ binds many proteins, including plasma proteins, adhesion and nuclear proteins, and inflammatory markers. Because TiO_2_ surfaces are generally negatively charged and hydroxylated in biological fluids, the binding is driven by a mix of electrostatic forces, hydrogen bonding, and hydrophobic interactions [24,25,26]. Some of these proteins contain specific motifs such as RKLPDA and related arginine- and lysine-rich sequences that anchor to TiO_2_ [27]. We have identified a TiO_2_-binding protein as a cell-surface protein in the bacterium *Rhodococcus* sp. [28], which was later shown to be homologous to the human mitochondrial enzyme DLDH [21] and used by us as a model for a TiO_2_-binding protein. DLDH was shown to bind TiO_2_ via well-characterized coordinative bonds [8].

In order to extend activity beyond ultraviolet illumination, extensive efforts have focused on modifying TiO_2_ through metal and nonmetal doping, defect engineering, and heterojunction formation. These approaches introduce mid-gap states, modify carrier lifetimes, and broaden optical absorption [4,17]. Experimental studies with doped TiO_2_ nanoparticles demonstrated efficient detoxification of organophosphorus compounds under both UV and visible light, highlighting the translational relevance of tailored electronic structures. In gas-phase catalysis, strong metal–support interactions transform TiO_2_ from a passive support into an active electronic partner, as shown for carbon monoxide oxidation, where interfacial coupling enhances oxygen activation and turnover [12,13,14].

At the nano–bio-interface, TiO_2_ rapidly acquires a biomolecular corona that defines its biological identity [29,30,31]. While such coronas are common to many nanomaterials, TiO_2_ is particularly effective at supporting coordinative binding, as exemplified by DLDH, which enables oriented and functionally persistent attachment of selected proteins. DLDH–TiO_2_ conjugates exemplify this behavior, maintaining stability in biological media and enabling predictable modulation of cell adhesion and signaling. RGD-modified constructs further demonstrate how molecular recognition motifs can be integrated with inorganic surfaces to control cellular engagement and localized redox responses [9,10,23]. These observations suggest a link between surface chemistry, protein orientation, and biological outcome, although further validation under physiological conditions remains necessary.

The versatility of TiO_2_ necessitates careful consideration of safety and biological impact. Particle size, crystalline form, and surface chemistry critically influence biodistribution and cellular responses. Reports of nanoparticle migration from implant sites highlight the importance of long-term evaluation [32], yet biological outcomes remain strongly context-dependent. Advanced characterization tools, including nanoscale flow cytometry and extracellular vesicle analysis, are increasingly essential for correlating physicochemical properties with functional and toxicological endpoints. These considerations further support the need for design strategies that account for size, surface chemistry, and biological environment to minimize unintended effects.

**Figure 1:**
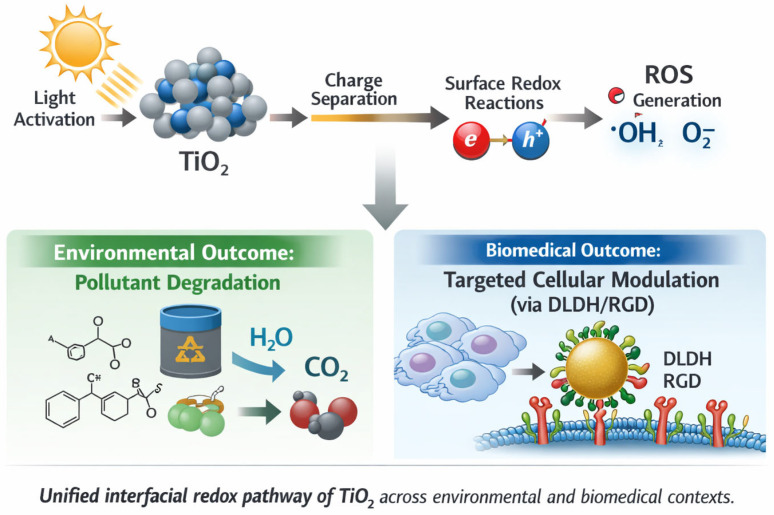
Unified interfacial redox pathway of TiO_2_ across environmental and biomedical contexts. Light excitation induces charge separation, leading to surface redox reactions and ROS generation. These processes diverge into environmental outcomes (pollutant degradation) and biomedical outcomes (targeted cellular modulation via protein-functionalized interfaces such as DLDH/RGD).

The mechanistic principles discussed above, including band alignment, defect-state modulation, and interfacial-coordination chemistry, are not restricted to biomedical interfaces. The same electronic processes govern photocatalytic ROS generation in environmental systems (Figure 1). Understanding how charge carriers are generated, transferred, and confined at TiO_2_ surfaces, therefore, provides a common foundation for both targeted redox modulation in biology and pollutant degradation in complex matrices. The following section examines these mechanisms from an environmental catalysis perspective.

## 3. Photocatalytic Mechanisms and Environmental Redox Engineering

Upon irradiation with photons exceeding the TiO_2_ band gap, electrons are promoted from the valence to the conduction band, generating electron–hole pairs whose fate determines photocatalytic efficiency [4,12]. Importantly, these redox processes are spatially confined to the TiO_2_ interface, where charge transfer and radical reactions occur within nanometer-scale proximity to the surface. This interfacial localization distinguishes heterogeneous photocatalysis from homogeneous oxidation systems and plays a key role in determining reaction selectivity and efficiency. Surface-trapped holes oxidize adsorbed water or hydroxide to yield hydroxyl radicals, whereas conduction-band electrons reduce molecular oxygen to superoxide, initiating a cascade that includes H_2_O_2_ and secondary ROS [33,34]. The competition between interfacial charge transfer and bulk recombination remains the primary limitation of quantum yield [1,35], emphasizing the importance of surface engineering. Hydroxyl radical formation proceeds via multiple routes, depending on surface chemistry and medium composition. The dominant pathway involves direct oxidation of surface hydroxyls by valence-band holes, while secondary contributions arise from H_2_O_2_ decomposition and superoxide-mediated reactions [34]. Doped TiO_2_ systems have demonstrated efficient •OH production under visible illumination when the electronic-structure is tailored appropriately, as shown in the detoxification of the organophosphate profenofos [14]. The surface-confined nature of these reactions limits radical diffusion, a property crucial for both environmental selectivity and potential biomedical translation. In aqueous systems, hydroxyl radicals generated at the interface exhibit extremely short diffusion lengths, reacting within nanometer-scale proximity to the surface. Consequently, degradation efficiency depends strongly on adsorption equilibria and surface coverage, distinguishing TiO_2_ photocatalysis from homogeneous advanced oxidation processes where radicals diffuse freely in bulk solutions. Recent advances in TiO_2_-based photocatalysis have highlighted the importance of precise electronic-structure control through emerging design strategies. Single-atom catalysis has gained attention, where isolated metal atoms anchored on TiO_2_ surfaces create well-defined active sites that enhance charge separation and catalytic selectivity. Oxygen vacancies have also been extensively studied as intrinsic defect states that introduce mid-gap energy levels, thereby extending light absorption and facilitating interfacial charge transfer. Furthermore, S-scheme heterojunctions have been proposed to enable efficient charge separation while preserving strong redox potentials, overcoming limitations associated with conventional type-II systems. These developments reflect a shift toward rational electronic structure engineering in TiO_2_-based photocatalysis [36,37,38,39,40].

ROS yield is governed by photon flux, adsorption equilibria, recombination rates, and availability of electron acceptors. Strong metal–support interactions markedly influence these parameters by promoting charge delocalization and extending carrier lifetimes. In SMSI systems, electronic coupling at the metal–oxide interface can induce charge redistribution, modify adsorption energies, and create interfacial states that facilitate oxygen activation. Such energetic coupling transforms TiO_2_ from a passive support into an electronically active partner, extending its functional role beyond simple photon absorption. In TiO_2_ microparticles employed for carbon monoxide oxidation, interfacial electronic coupling enhanced oxygen activation and catalytic turnover [13], illustrating how SMSI can modulate redox efficiency under ambient conditions. From an application perspective, these parameters are closely linked to energy efficiency, which represents a critical factor in evaluating scalability. Metrics such as electrical energy per order (EEO) and photon utilization efficiency are increasingly used to assess practical performance beyond laboratory-scale conditions.

**Figure 2:**
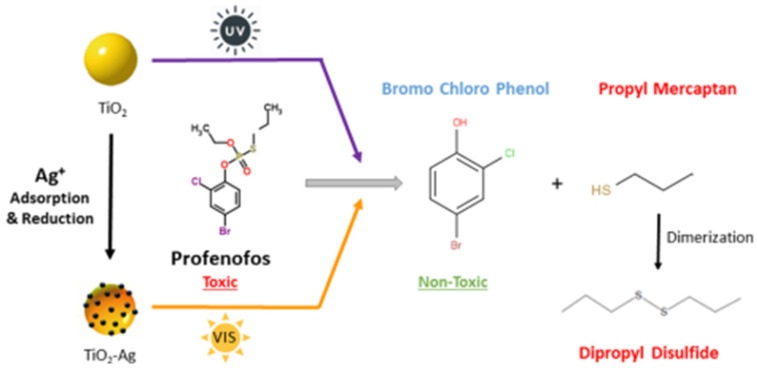
Photocatalytic detoxification pathway of the organophosphate profenofos on Ag-modified TiO_2_. Schematic representation of adsorption and reduction of Ag^+^ on TiO_2_, followed by UV and visible light activation, leading to bond cleavage in profenofos. The process yields bromo-chlorophenol, classified as non-toxic, and propyl mercaptan, which undergoes dimerization to dipropyl disulfide, demonstrating conversion of a toxic pesticide into less harmful products. Bromo-chlorophenol is considered less toxic than profenofos due to the absence of organophosphate functional groups responsible for acute toxicity. Reprinted with permission from the Journal of Physical Chemistry A.

The non-selective oxidative capacity of •OH underlies TiO_2_-based degradation of pesticides and volatile pollutants. Photocatalytic cleavage of P–O and C–S bonds in organophosphates has been documented under UV and visible light, with kinetics strongly dependent on dopant composition and matrix effects [14]. An illustrative example of this mechanism is shown in Figure 2, where photocatalytic activation of Ag-modified TiO_2_ under UV and visible illumination induces cleavage of the organophosphate pesticide profenofos into the less toxic intermediates bromo-chloro phenol and propyl mercaptan, followed by dimerization of the thiol product to dipropyl disulfide.

These studies demonstrate the feasibility of in situ detoxification without secondary reagents, a key advantage for field-deployable remediation. However, real-world matrices introduce significant constraints. These limitations emphasize the gap between controlled experimental systems and complex environmental conditions, underscoring the need for standardized evaluation protocols and realistic benchmarking of photocatalytic performance. Natural organic matter, bicarbonate ions, and elevated ionic strength can act as radical scavengers or compete for active sites, substantially reducing effective degradation rates compared to model laboratory systems. In biological contexts, ROS function both as signaling mediators and potential cytotoxins. This dual role highlights the importance of controlling not only ROS generation but also their spatial and temporal distribution, particularly when translating photocatalytic principles into biomedical applications. Cancer cells frequently operate near oxidative thresholds, rendering them sensitive to localized redox perturbation [41,42,43]. Protein–TiO_2_ hybrids illustrate how photocatalytic principles can be adapted for spatially confined modulation: DLDH-based constructs coupled to TiO_2_ have been shown to generate ROS upon illumination while retaining molecular targeting through RGD motifs [9,22,23]. Such systems represent proof-of-concept frameworks rather than established therapies, highlighting the need for precise control of dose and localization. Importantly, extrapolation from controlled gas-phase or aqueous degradation studies to complex environmental scenarios requires cautious interpretation, as mass transfer limitations and fluctuating irradiation conditions can significantly alter apparent kinetics. Recent reports also highlight the importance of nanostructure design and surface functionalization in modulating biological responses and therapeutic performance of TiO_2_-based systems [38,40,44].

At the nano–bio-interface, adsorbed proteins and local dielectric properties reshape photocatalytic behavior. Coordinatively bound DLDH retains stability over repeated activation cycles [8,9,23], enabling reproducible redox responses. Quantitative tools such as high-resolution flow cytometry support analysis of particle–cell interactions and vesicle-mediated signaling, providing mechanistic insight into how surface chemistry translates into biological outcome.

While the preceding discussion focused on fundamental charge dynamics and radical formation pathways, practical deployment of TiO_2_ technologies depends on how these mechanistic parameters translate into reactor-scale performance. The following section. Therefore, shifts from electronic and interfacial processes to engineering considerations governing real-world environmental applications.

## 4. Environmental Applications of TiO_2_-Based Systems

The mechanistic principles described above directly inform the design and operation of photocatalytic systems under real-world conditions, as the accumulation of persistent organic pollutants in air and water has driven extensive exploration of TiO_2_ as a sustainable photocatalyst. Unlike conventional oxidants, TiO_2_ enables in situ mineralization of contaminants using light as the primary energy input [4,12]. From an engineering perspective, the practical implementation of TiO_2_-based systems depends not only on intrinsic catalytic activity but also on reactor design, light delivery, and system-level optimization. Hydroxyl radicals generated at the catalyst surface promote stepwise cleavage of complex molecules toward CO_2_ and inorganic ions, reducing the formation of secondary toxic intermediates. Organophosphorus pesticides and nerve-agent surrogates constitute a major class of targets due to their stability and toxicity. Photocatalytic systems based on doped TiO_2_ have demonstrated rapid degradation through cleavage of P–O and P–S bonds under UV and visible illumination [14]. These studies highlight two critical aspects: first, the ability to neutralize highly active molecules directly at the contamination site, and second, the dependence of kinetics on matrix composition, pH, and competing scavengers, factors that often limit translation from model solutions to real waters. However, translating these findings into real-world applications requires careful consideration of system complexity, including competing reactions, mass transfer limitations, and variable environmental conditions.

In gas-phase applications, TiO_2_ participates actively in heterogeneous reactions rather than serving solely as an inert support. Strong metal–support interactions modify the electronic structure of both the oxide and supported metals, enhancing oxygen activation and turnover. This behavior was exemplified in carbon monoxide oxidation, where TiO_2_ microparticles facilitated efficient catalytic cycles under ambient conditions [13]. Such findings are directly relevant to the design of catalytic filters and indoor air purification units. The reliance of pristine TiO_2_ on ultraviolet light has motivated the development of visible-active systems through doping and heterojunctions [45]. Long-term stability of doped or composite TiO_2_ systems must also be considered, as dopant leaching, surface restructuring, or photocorrosion may gradually alter electronic properties and reduce catalytic performance over extended operation. Hybrid composites incorporating carbon materials or plasmonic nanoparticles improve charge separation and broaden absorption spectra [46,47,48]. Importantly, performance reported under ideal laboratory conditions often overestimates field efficacy; natural organic matter, turbidity, and ionic strength can strongly quench ROS or block active sites. Standardized metrics such as electrical energy per order (EEO), photon utilization efficiency, and catalyst stability are essential for realistic comparison and evaluation of large-scale feasibility. Long-term deployment requires solutions to catalyst recovery and stability. Suspended nanoparticles maximize surface area but complicate separation and raise concerns of release, whereas immobilized configurations reduce dispersion at the cost of mass transfer limitations. Suspended nanoparticle systems offer maximal surface area and enhanced mass transfer but require post-treatment separation and raise concerns regarding nanoparticle release. In contrast, immobilized TiO_2_ coatings enable easier recovery and improved operational safety yet often suffer from reduced active surface exposure and diffusion limitations. The choice between configurations, therefore, represents a trade-off between catalytic efficiency and practicality in engineering. Modern reactor design increasingly integrates catalyst architecture with hydrodynamics and irradiation geometry, reframing TiO_2_ remediation as a systems-engineering challenge rather than a materials problem alone [47]. Key engineering challenges include achieving uniform light distribution, minimizing energy losses, and maintaining catalytic performance over extended operational cycles. Scale-up introduces additional challenges, including non-uniform light penetration in larger reactors, catalyst deactivation over repeated cycles, and accumulation of intermediate products that may inhibit surface activity. Addressing these challenges requires integrated optimization of materials, reactor configurations, and operating conditions rather than focusing on catalyst performance alone. These factors frequently lead to discrepancies between laboratory-scale performance and field implementation. Key design parameters include catalyst loading, photon flux distribution, surface-to-volume ratio, flow dynamics, and residence time. Performance metrics should be evaluated not only in terms of degradation rate constants but also energy efficiency, catalyst stability, and resistance to fouling under realistic operational conditions.

A conceptual parallel exists between environmental and biomedical uses of TiO_2_: in both, hydroxyl radicals serve as the active species, yet the design goals diverge from maximal oxidation toward spatial control. Knowledge gained from advanced oxidation processes informs strategies for localized redox modulation in biological settings [34,49,50], illustrating the cross-disciplinary value of environmental photocatalysis, while emphasizing the need for controlled and application-specific redox design.

## 5. Biomedical Applications of TiO_2_

Titanium dioxide has long been utilized in implants as a corrosion-resistant oxide layer; however, it is now recognized as an active bio-interface that shapes protein adsorption and cellular signaling [51,52,53]. Surface hydroxylation and charge distribution regulate integrin clustering and cytoskeletal organization, enabling TiO_2_ to modulate biological responses beyond passive structural support. This perspective aligns with contemporary nanomedicine, where inorganic materials function as controllable redox and signaling platforms [54,55,56]. A conceptual advance in TiO_2_ biotechnology is the exploitation of moonlighting proteins as functional bridges. DLDH, classically a mitochondrial enzyme, exhibits additional activities, including metal binding [8], DNA interaction [9,10,23] and redox modulation [11]. These properties enable DLDH to anchor to TiO_2_ through coordinative interactions while retaining functional flexibility, providing a modular route to couple inorganic surfaces with biological specificity. Detailed mapping revealed that DLDH attaches to TiO_2_ via discrete metal-chelating residues rather than nonspecific physisorption [8,9,10,23]. The resulting orientation supports long-term stability in biological media and forms the basis for predictable biofunctionalization. Such semi-covalent interfaces differentiate TiO_2_ from many alternative substrates where protein activity is rapidly lost.

Integrin receptors, particularly αvβ3/β5, are overexpressed in tumors and angiogenic tissues, making them established molecular targets [57,58,59]. Incorporation of RGD motifs into DLDH constructs enables selective recognition, converting the protein into a targeting module. On TiO_2_ surfaces, RGD–DLDH conjugates enhanced osteogenic cell adhesion and spreading, illustrating how molecular cues can be integrated with inorganic materials to guide tissue responses [23].

Photodynamic therapy relies on light-triggered ROS generation to induce localized cellular damage [60,61,62]. TiO_2_–DLDH-RGD conjugates provide a platform in which integrin targeting and photocatalytic activation are combined, enabling spatially confined redox perturbation upon illumination [9]. Although such systems remain at a proof-of-concept stage and require further validation under physiologically relevant conditions.

**Figure 3:**
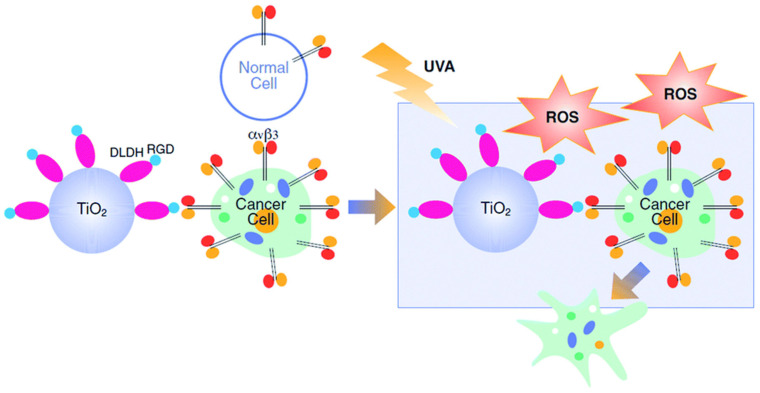
Integrin-targeted ROS generation by RGD–DLDH-functionalized TiO_2_ nanoparticles. Schematic representation of selective binding of DLDH–RGD-modified TiO_2_ to αvβ3 integrins overexpressed on cancer cells. Upon UVA illumination, the conjugates produce localized reactive oxygen species, inducing redox stress in malignant cells while minimizing effects on neighboring normal cells, illustrating a proof-of-concept for spatially confined photodynamic modulation. Reprinted with permission from the Royal Society of Chemistry Advances.

These systems represent preclinical proof-of-concept frameworks that illustrate the potential of externally controllable TiO_2_ interfaces, while requiring further validation of selectivity, dosing, and safety. Key translational challenges include limited light penetration in tissues, potential loss of surface functionalization under protein-rich biological conditions, and difficulties in maintaining reproducible activity at clinically relevant scales. Cancer cells often operate near oxidative thresholds, rendering them susceptible to additional ROS elevation [41,42,43]. Integrin-directed TiO_2_ constructs exploit this vulnerability by localizing redox activity to defined cellular compartments [22]. Such strategies complement broader efforts to modulate tumor metabolism rather than acting as stand-alone therapies. The conceptual basis of this targeted approach is illustrated in Figure 3, where RGD-modified DLDH–TiO_2_ conjugates selectively associate with αvβ3 integrins on cancer cells and, upon UVA illumination, generate localized reactive oxygen species while sparing adjacent normal cells.

In orthopedic and dental applications, rapid osteointegration is essential. DLDH–RGD functionalization of TiO_2_ surfaces promoted adhesion and cytoskeletal organization of bone-forming cells [23], suggesting routes to bioactive coatings that act through molecular recognition rather than topography alone. Evaluation of these interfaces requires advanced analytics. High-resolution flow cytometry and extracellular vesicle profiling enable quantitative assessment of nano–bio communication and oxidative signaling, linking material properties with cellular outcomes.

Despite promising proof-of-concept demonstrations, a substantial translation gap remains between in vitro redox modulation and clinical implementation. Key unresolved issues include quantitative control of ROS dose under physiologically relevant light penetration depths, stability of functionalized surfaces under protein-rich in vivo conditions, and reproducible large-scale manufacturing of defect-engineered TiO_2_ with defined electronic properties. These limitations highlight the importance of integrating material design, targeting strategies, and controlled-activation mechanisms to enable clinically relevant applications. Despite promising proof-of-concept demonstrations, a substantial translation gap remains between in vitro findings and clinical implementation. Key challenges include limited light penetration in biological tissues, instability of surface functionalization under physiological conditions, and difficulties in achieving reproducible large-scale synthesis. Addressing these limitations will require integration of material design, targeting strategies, and controlled-activation mechanisms.

## 6. Toxicological and Biocompatibility Considerations

Nanomaterial safety cannot be extrapolated from bulk chemistry alone; biological responses depend on size, surface state, and transformation in situ [63,64,65]. Accordingly, there is a growing shift from passive toxicity assessment toward proactive safety-by-design strategies, in which physicochemical parameters are deliberately tuned to minimize adverse biological effects.

TiO_2_ exemplifies this duality: it is widely used in implants and consumer products, yet nanoscale variants may elicit context-dependent effects. Thus, TiO_2_ should be regarded as a family of materials rather than a single entity. Accordingly, safety and biological outcomes must be evaluated in a context-dependent manner, rather than assuming uniform behavior across all TiO_2_ forms. Bulk TiO_2_ layers on implants are generally stable, poorly mobile, and biologically inert under physiological conditions. In contrast, nanoscale TiO_2_ exhibits increased surface area, higher defect density, and greater potential for cellular internalization, all of which significantly alter biological interaction profiles.

At the cellular level, excessive ROS may damage lipids and nucleic acids, whereas controlled levels participate in signaling. Biological outcomes follow a dose–response continuum in which localized redox modulation can be beneficial while widespread activation may trigger inflammation. DLDH-based systems illustrate this balance and highlight how controlled biofunctionalization may be used to achieve localized redox activity while limiting nonspecific effects [11]. This reinforces the need to engineer systems that enable spatial and temporal control of ROS generation, rather than maximizing oxidative capacity [11].

Particle size and surface chemistry influence translocation from implant sites to systemic compartments. A prospective study combining in vivo and imaging approaches reported possible neurological effects associated with titanium-derived particles [32]. These findings emphasize the need for life-cycle assessment, though they do not imply uniform risk across all TiO_2_ forms. Successful implantation demands minimal chronic inflammation and stable osteointegration. DLDH–RGD-modified surfaces enhanced cellular adhesion without intrinsic cytotoxicity in the absence of external activation [23], illustrating how functionality can remain dormant under physiological conditions. Comprehensive evaluation requires multidimensional characterization, including aggregation state, corona composition, oxidative thresholds, and immune responses. Techniques such as nanoscale flow cytometry provide early indicators of perturbation. From a design perspective, these observations suggest that controlling particle size distribution, crystalline phase composition, and surface functionalization may provide practical routes to reduce nonspecific biodistribution and improve safety profiles. Recent studies further demonstrate that surface modification, polymer integration, and membrane-based systems can influence nanoparticle stability, transport, and biological interaction profiles [44,66,67]

Regulatory agencies increasingly adopt tailored frameworks for nanomaterials. Current guidance emphasizes material characterization, life-cycle analysis, and post-market surveillance for nano-enabled products initiatives further aim to harmonize testing strategies for manufactured nanomaterials [68]. These evolving frameworks further support the implementation of safety-by-design principles as a central component in the development of nano-engineered TiO_2_ systems. Key principles for safer TiO_2_ systems include spatial confinement of ROS, prevention of uncontrolled release, stable anchoring of biomolecules, and advanced monitoring. Importantly, parameters such as particle size distribution, crystalline phase ratio, defect density, and surface functionalization directly influence oxidative potential, protein interaction patterns, and immune recognition. As a result, laboratory-scale efficiencies often overestimate field performance, emphasizing the need for testing under environmentally relevant conditions. Risk assessment must therefore integrate physicochemical characterization with biological outcome measures rather than rely on nominal material identity. These guidelines support translation toward clinically and environmentally acceptable technologies. Nonetheless, long-term epidemiological data remain limited, and cautious interpretation is warranted when extrapolating short-term experimental findings to chronic exposure scenarios. Overall, integrating safety considerations at the design stage, rather than as a post hoc evaluation, is expected to play a critical role in enabling the safe and effective translation of TiO_2_-based technologies.

## 7. Emerging Directions and Future Perspectives

Recent advances in TiO_2_ research highlight a transition from passive photocatalysts toward systems designed for controlled and context-dependent redox activity. Future developments are likely to focus on systems that enable controlled activation in response to external stimuli such as light, local chemical environment, or biomolecular interactions [69,70,71]. Emerging studies further indicate that integrating photocatalysis with advanced materials platforms, including membranes and hybrid composites, may enable improved scalability and functional performance [44,66,72].

TiO_2_ surfaces functionalized with molecular switches or moonlighting proteins offer conditional activity. DLDH–TiO_2_ constructs illustrate how externally triggered ROS can be generated while remaining inert at baseline [9,23]. Such concepts are relevant for infection-resistant implants and localized redox modulation. Composites with carbon materials or plasmonic nanoparticles may enhance charge separation and broaden spectral response. Key challenges include maintaining charge separation efficiency under visible or near-infrared activation, preventing recombination losses, and preserving sufficient redox potential for effective catalytic or biological activity. However, shifting activation toward the visible or near-infrared range while preserving catalytic efficiency remains a fundamental physical challenge. Band-gap narrowing must be balanced against increased recombination and potential loss of redox driving force. These multimodal systems enable simultaneous catalytic detoxification and bio-interfacing, provided that ROS profiles are carefully tuned. Environmental and biomedical TiO_2_ applications share common redox mechanisms; however, they differ significantly in their requirements for selectivity, localization, and control processes. Knowledge retained from advanced oxidation processes informs design of biologically compatible systems, while targeting strategies enrich environmental technologies [34,49]. Progress will rely on analytical tools such as high-resolution flow cytometry and computational modeling to predict nano–bio interactions [56]. Future research should integrate quantitative ROS mapping in complex media, standardized defect-state characterization, and long-term stability testing under cyclic irradiation. Bridging computational band-structure modeling with biological outcome measurements will be essential to translate electronic design into predictable physiological behavior. Systems offering spatially confined and externally controlled activity present favorable safety profiles. Alignment with regulatory frameworks will be essential for clinical and industrial adoption. Manufacturing reproducibility, defect-state control at scale, and batch-to-batch electronic consistency represent non-trivial industrial hurdles. Regulatory approval will likely require integrated physicochemical and biological characterization strategies tailored specifically to nano-engineered oxide systems. Advancing TiO_2_-based systems will require coordinated progress in three areas: (i) precise control of electronic structure to enable efficient and tunable redox activity, (ii) development of stable and reproducible surface functionalization strategies for biological interfaces, and (iii) integration of material properties with reactor- or system-level design to ensure practical applicability.

Three central challenges define the next stage of TiO_2_ development. First, the achievement of an efficient visible-light activation within biologically relevant penetration depths without compromising electronic stability. Second, ensuring spatial confinement of ROS to prevent off-target oxidative damage. Third, establishment of reproducible and scalable synthesis protocols that maintain controlled defect density and interfacial functionality across production batches.

TiO_2_ remains a relevant platform due to its adaptability and well-characterized properties; however, its successful application will depend on the ability to achieve controlled, reproducible, and application-specific redox behavior. When combined with moonlighting proteins and targeting motifs, it forms an intelligent platform linking sustainability with precision medicine.

## 8. Summary and Conclusions

Titanium dioxide (TiO_2_) is a well-established material whose applications span environmental photocatalysis and biomedical interfaces span environmental photocatalysis. Across both domains, a common physicochemical core emerges: photoinduced charge separation, surface-mediated redox reactions, and the generation of reactive oxygen species. These mechanisms enable degradation of hazardous pollutants in air and water while, in biological contexts, allowing spatially confined modulation of cellular processes. This review highlights shared physicochemical mechanisms governing TiO_2_ activity across environmental and biological systems, environmental photocatalysis, and biomedical redox modulation within a shared interfacial engineering paradigm, rather than treating them as independent application domains.

Advances in materials engineering, including doping, heterojunction design, and strong metal–support interactions, have markedly improved catalytic efficiency and expanded activity into the visible range. At the same time, the ability of TiO_2_ to engage in coordinative interactions with biomolecules has enabled the development of bio functional interfaces based on coordinative interactions. Moonlight proteins such as dihydrolipoamide dehydrogenase illustrate how biological components can be stably integrated with inorganic surfaces to confer targeting, orientation, and controllable redox activity.

Biomedical explorations of TiO_2_ highlight its potential as a controlled redox activation. RGD-mediated integrin recognition and light-triggered ROS generation demonstrate the feasibility of translating photocatalytic mechanisms into localized biological contexts, including targeted therapeutic approaches and enhanced osteointegration. Toxicological evidence further emphasizes that biological outcomes are context-dependent; are governed by particle size, surface chemistry, and exposure scenarios; and must be evaluated through advanced analytical and regulatory frameworks. Significant open challenges remain, including limited light penetration in living tissues, incomplete control over spatial ROS confinement, and uncertainties regarding long-term nanoparticle fate under chronic exposure scenarios.

From an environmental perspective, TiO_2_ continues to be widely used in photocatalytic environmental remediation. However, practical implementation requires realistic performance benchmarking in complex matrices and sustained catalyst stability under operational stress. Photocatalytic detoxification of organophosphates and gas-phase pollutants demonstrates its in situ applicability as a treatment without secondary reagents. Real-world deployment, however, requires reactor-level design, immobilization strategies, and standardized performance metrics to bridge the gap between laboratory demonstrations and field applications.

Overall, TiO_2_ should be regarded as a material system whose properties depend on surface chemistry, electronic structure, and application context, and whose behavior is dictated by interface chemistry. Future applications will depend on achieving controlled, reproducible, and application-specific redox behavior, whose value depends on precise electronic control, spatial redox regulation, and context-aware safety design. These domains should not be viewed as interchangeable applications, but rather as systems governed by shared mechanisms yet distinct performance criteria.

## Data Availability

No new data were created or analyzed in this study. Data sharing is not applicable to this article.
